# Editorial: Nature-based learning and development: maximizing the returns on investment, volume II

**DOI:** 10.3389/fpsyg.2023.1304644

**Published:** 2023-10-16

**Authors:** Ming Kuo, Ulrich Dettweiler, Andrea Faber Taylor, Catherine Jordan, Nancy M. Wells

**Affiliations:** ^1^Landscape and Human Health Laboratory, Department of Natural Resources and Environmental Sciences, University of Illinois at Urbana-Champaign, Champaign, IL, United States; ^2^Department of Cultural Studies and Languages, Faculty of Arts and Education, University of Stavanger, Stavanger, Norway; ^3^Department of Crop Sciences, University of Illinois at Urbana-Champaign, Champaign, IL, United States; ^4^Department of Pediatrics, University of Minnesota, Saint Paul, MN, United States; ^5^Children and Nature Network, University of Minnesota, Minneapolis, MN, United States; ^6^Department of Design and Environmental Analysis, Cornell University, Ithaca, NY, United States

**Keywords:** nature, nature-based learning, learning, development, return on investment

The evidence is in: *experiences with nature promote learning and development*. Findings from upwards of a thousand studies—from fields as disparate as leisure studies, education, landscape architecture, public health, and psychology, and from wilderness backpacking to plants in a preschool to lessons on frogs—show that experiences with nature contribute to learning and healthy development (e.g., Research Topic: The Natural World as a Resource for Learning and Development: From Schoolyards to Wilderness; for review, see Kuo et al.). And in head-to-head comparisons, “nature-based” learning outperforms conventional classroom learning (e.g., Wells et al., 2015). It's time for “nature-based learning” (NBL) to move from research into standard practice.

This Research Topic aims to support that move. We propose that researchers can help move NBL into practice by incorporating a return on investment (ROI) perspective. The articles here illustrate several ROI Best Practices^1^:

- CONVEY THE “ROI.” Capturing both costs and benefits of a nature-based intervention in a single sentence helps practitioners decide whether an intervention is feasible and worth implementing, e.g., “a difference of 20% points of green space… is associated with an over 10% lower probability of children using ADHD medication” (de Vries and Verheij). See Traynor et al.'s Figure 1 for a graphical representation of ROI.
- DESCRIBE THE INVESTMENT. Describing interventions in detail (e.g., Traynor et al.) makes it easier for practitioners to adopt them successfully, with less trial and error. Quantifying labor, materials, and other costs aids decision-making about, and preparation for, adoption.
- IDENTIFY THE MINIMUM INVESTMENTS for a desired return. When the minimum investment is small, this information can encourage adoption; when the minimum investment is large, this information can avert underinvestment. Ernst et al. find that incorporating a few nature-based lessons in a traditional curriculum is enough to yield multiple, important benefits, but that a full nature preschool experience may be needed to specifically boost children's initiative.
- IDENTIFY KEYS TO DESIRED RETURNS. Not all intervention components contribute to desired outcomes; de Vries and Verheij find the relationship between residential nature and ADHD medication usage may depend primarily on overall “greenspace” and not particular “green elements.” Identifying the “active ingredients” of an intervention helps practitioners know which must be replicated precisely and which ones practitioners might be able to forgo. Similarly, information about mechanisms helps practitioners know how they might maximize returns. Mateer suggests nature's eudaimonic benefits may stem from experiences of awe and solitude, providing considerably more direction to park designers than the simple exhortation to “provide nature.”
- CONSIDER EQUITY, a common and important return on nature interventions. Examining impacts of an intervention separately for different groups enables us to see if a given intervention reduces, exacerbates, or replicates existing inequities—de Vries and Verheij and Hartley et al. do, and find an “equigenic” effect.
- STUDY THE UNDERSTUDIED to provide scientific guidance for policy and practice where little exists. Cosco et al. focus on an understudied population (childcare centers serving low-income families) and an outcome of special importance to that population (fruit and vegetable consumption).
- CONSIDER CONTEXT. Two kinds of contextual factors are important for practitioners: “prerequisites” (conditions needed for an intervention to be fully implemented) and “moderators” (situational factors likely to affect the returns from an intervention, once implemented). “Prerequisites” tell practitioners where an intervention is and isn't feasible—for specific examples, see Beauchamp et al.'s facilitating and limiting factors and Traynor et al.'s “requirements.” “Moderators” tell practitioners whether returns are likely to be lower (or higher) in their particular context, and why—see Ellinger et al.'s discussion of potential moderators that might explain why prosocial outcomes typically found in nature failed to appear in their specific context.

The articles here not only illustrate best practices for guiding widespread adoption of NBL but also help us imagine potential returns of that transformation. What if education and environmental design were reshaped to take advantage of nature's powerful effects on learning and development?

## Nature-based education

If the nature-based interventions here were extended to multiple developmental stages from preschool to college and adopted at the population level, we might see larger, lifelong, population-level benefits.

A HEALTHIER POPULATION

- If the hands-on gardening in childcare in Cosco et al.'s study was widely adopted and extended into elementary school and beyond, we might see lifelong healthy diets, reducing obesity and disease.
- If the impacts of nature experiences on psychological resilience in both preschoolers and college students found in Ernst et al. and Rakow and Ibes, respectively, were reinforced in K-12 education, we might see future generations better at coping with adversity.
- If nature prescriptions (Rakow and Ibes) were extended to younger-than-college ages and widely adopted, we might see population gains in mental health across the lifespan.

A BETTER-EQUIPPED CITIZENRY—a population with 21st-century skills, more inclined and better prepared to tackle the largest challenges of our time:

- Time in nature appears to foster 21st-century skills and dispositions such as leadership, initiative, communication, collaboration, critical thinking, and creativity (Mann et al.; Ernst et al.; Schilhab).
- Contreras and Krasny find that nature-based projects can empower children as young as pre-k and kindergarteners to be environmental stewards, helping them recognize their capacity to meaningfully contribute to their communities.
- If nature-based science education were the norm, we might expect a more scientifically literate workforce and citizenry, with a stronger foundation in environmental education (for review, see Schilhab).

A MORE JUST AND INCLUSIVE SOCIETY

- Nature-based interventions are often especially effective for underserved populations; see de Vries and Verheij (residential greenspace and low-SES populations) and Hartley et al. (nature-based education and linguistically diverse learners).
- Natural settings seem to afford more inclusive pedagogy—e.g. Beauchamp et al. report teachers incorporating First Nations and Indigenous cultural practices.
- Nature-based interventions can address important outcomes among marginalized populations (e.g. Cosco et al.).

One of the most striking emergent themes in this collection is the difference between two iterations of NBL. NBL 1.0 requires only bringing natural materials into the classroom or bringing lessons outside, and delivers greater *learning*, whereas NBL 2.0 requires a major change in pedagogy, but fosters *development*, including the 21st-century skills that conventional pedagogy and NBL 1.0 fall short on delivering. Chawla points out that such benefits as autonomy, competence, relatedness, and “eudaimonic thriving” require one to be an “active agent” in nature, rather than a passive recipient. Consistent with Chawla's conception, outcomes like communication, flexibility, problem-solving, and leadership/initiative appear almost exclusively in student-centered or student-driven settings in the other articles in this collection (Beauchamp et al.; Contreras and Krasny; Mann et al.; Traynor et al.; Ernst et al.; Hartley et al.). Schilhab discusses barriers to less structured, more autonomous nature experiences in science learning. In these settings, students are “active agents” and teachers are flexible and responsive.

## Nature-based environmental design

Not surprisingly, when we use the findings here to imagine the potential returns of nature-based environmental design, the same themes emerge: health, better-equipped adults, and reduced inequity. If all neighborhoods contained sufficient greenspace, we might see not only less need for ADHD medications but improved academic achievement and greater earnings through the lifecourse for that population—with especially large effects in the neighborhoods most deprived of greenspace (de Vries and Verheij). If parks were designed with “zones” for both outdoor recreation and experiences of awe and solitude, as recommended by Mateer, we might see both hedonic and eudaimonic benefits.

## Conclusion

In this Research Topic of 10 empirical articles, one review, and two conceptual pieces, we have attempted to demonstrate the value of a ROI approach and to highlight resulting evidence for best practices to guide wide-spread adoption of NBL. These 13 articles spark a vision for education and environmental design reimagined to leverage the significant demonstrated impact of nature on both learning (NBL 1.0) and development (NBL 2.0).

## Author contributions

MK: Conceptualization, Writing—original draft, Writing—review and editing. UD: Conceptualization, Writing—original draft, Writing—review and editing. AF: Conceptualization, Writing—original draft, Writing—review and editing. CJ: Conceptualization, Writing—original draft, Writing—review and editing. NW: Conceptualization, Writing—original draft, Writing—review and editing.

## Footnotes

^1^For more ideas on how researchers can increase the impacts of their work on policy and practice, see Kuo (2002).
